# Patient-Level *KRAS* G12C Prevalence among Hispanic/Latino and Non-Hispanic White Patients in Real-world Clinicogenomic Cohorts

**DOI:** 10.1158/2767-9764.CRC-26-0322

**Published:** 2026-09-07

**Authors:** Daniel F. Pilco-Janeta, Myriam De la Cruz-Puebla, Daisy R. Guamán-Pilco, Diego Montenegro, William Miranda

**Affiliations:** 1 Latin American Institute of Precision Oncology (CancerLat), Quito, Ecuador.; 2 Department of Pathology, Brigham and Women’s Hospital, Harvard Medical School, Boston, Massachusetts.; 3 Department of Internal Medicine, Health Sciences Faculty, Technical University of Ambato, Ambato, Ecuador.; 4 Atlantic Fellows for Equity in Brain Health, University of California, San Francisco (UCSF), San Francisco, California.; 5 Department of Oncology, Oncology Institute of Eastern Bolivia, Santa Cruz, Bolivia.; 6 Department of Health Services, Ministry of Public Health, Managua, Nicaragua.

## Abstract

KRAS G12C is a treatment-defining biomarker in non–small cell lung cancer (NSCLC), particularly lung adenocarcinoma. Hispanic/Latino (H/L) patients remain undercharacterized in precision oncology datasets, and patient-level KRAS G12C prevalence has not been consistently quantified using strict ethnicity definitions. We analyzed MSK-CHORD as the initial reference cohort and American Association for Cancer Research (AACR) GENIE after excluding the Memorial Sloan Kettering Cancer Center contributing center. H/L required explicit H/L-origin annotation; non-Hispanic White (NH-W) required White race with explicit non-Hispanic ethnicity. Primary analyses compared patient-level KRAS G12C prevalence in mutually exclusive lung adenocarcinoma and non–lung adenocarcinoma NSCLC. In lung adenocarcinoma, KRAS G12C prevalence was lower in H/L than NH-W patients in MSK-CHORD [7.9% vs. 15.4%; odds ratio (OR), 0.47; 95% confidence interval (CI), 0.30–0.74; *P* < 0.001] and GENIE (7.2% vs. 15.1%; OR, 0.44; 95% CI, 0.33–0.58; *P* < 0.0001). In non–lung adenocarcinoma NSCLC, differences were smaller and not significant in MSK-CHORD (4.8% vs. 5.1%; OR, 0.94; *P* = 1) or GENIE (4.9% vs. 6.7%; OR, 0.73; *P* = 0.461). In MSK-CHORD lung adenocarcinoma, adjustment for smoking shifted the OR from 0.50 to 0.68. H/L patients had lower patient-level KRAS G12C prevalence than NH-W patients with lung adenocarcinoma across two real-world clinicogenomic cohorts. The finding was histology specific and attenuated after smoking adjustment, supporting a biomarker-yield interpretation.

**Significance:**

H/L patients remain undercharacterized in real-world precision oncology datasets. This study provides patient-level, histology-specific estimates of KRAS G12C prevalence among H/L and NH-W patients using strict race/ethnicity definitions, coverage-aware denominators, and two large clinicogenomic cohorts. These findings inform biomarker screening yield in lung adenocarcinoma and support more rigorous, population-aware evaluation of actionable alterations in real-world oncology datasets.

## Introduction

*KRAS* G12C is a clinically actionable oncogenic alteration with direct therapeutic relevance in *KRAS*-mutant cancer. Unlike many *KRAS* substitutions, the cysteine residue generated by G12C enables allele-specific covalent inhibition, making this variant a biomarker with direct treatment implications (1, 2). Its clinical relevance is greatest in non–small cell lung cancer (NSCLC), particularly lung adenocarcinoma, where *KRAS* G12C represents a recurrent driver event and occurs within a molecular background frequently shaped by tobacco exposure and co-alterations in genes such as *STK11*, *KEAP1*, *SMARCA4*, and *TP53* (3, 4). In previously treated advanced NSCLC, *KRAS* G12C inhibitors have demonstrated clinical activity, supporting the role of this alteration as a treatment-defining biomarker (5, 6). Estimating the prevalence of *KRAS* G12C across patient populations is therefore relevant not only for descriptive epidemiology but also for molecular screening yield, therapeutic eligibility, clinical trial design, and interpretation of real-world precision oncology cohorts.

*KRAS* G12C prevalence is not expected to be uniform across all patients with cancer. Its frequency depends strongly on tumor lineage and is particularly enriched in lung adenocarcinoma compared with many other tumor types. Within NSCLC, *KRAS* G12C also exists within a broader tobacco-associated molecular phenotype that differs from other oncogenic driver states, including *EGFR*-mutant disease. These biologic and clinical dependencies are important when evaluating differences across demographic groups; an observed difference in *KRAS* G12C frequency may reflect the joint distribution of histology, smoking exposure, sequencing context, and co-mutational structure rather than a single isolated mechanism. Prior studies have reported differences in the genomic landscape of lung adenocarcinoma across racial and ethnic groups, including variation in *KRAS* and *EGFR* alterations (7).

Hispanic/Latino (H/L) patients remain incompletely characterized in large precision oncology datasets relative to non-Hispanic White (NH-W) patients. Although race and ethnicity are increasingly available in clinicogenomic resources, these variables are social and demographic classifications rather than direct measures of genetic ancestry. Analyses using H/L and NH-W categories therefore require careful interpretation and rigorous denominator definition. This is especially important for targeted sequencing datasets, in which gene-level ascertainment can vary by assay, institution, and cohort composition. In Hispanic and Latin American NSCLC cohorts, *KRAS* G12C prevalence has been reported to vary across regions and population substructures, supporting the need for carefully defined cohort-level analyses (8).

Prior studies have reported racial and ethnic differences in lung cancer driver alterations, including *EGFR* and *KRAS*. However, allele-specific *KRAS* G12C prevalence in H/L compared with NH-W patients has not been consistently quantified using strict race/ethnicity definitions, mutually exclusive lung histology strata, patient-level aggregation, and coverage-aware gene denominators (9, 10). This gap is clinically relevant because *KRAS* G12C is now a treatment-defining alteration, and differences in its prevalence may affect the expected number of eligible patients identified through routine molecular profiling. American Association for Cancer Research (AACR) GENIE and MSK-CHORD provide complementary real-world clinicogenomic resources for this type of analysis (11–13).

In this study, we evaluated patient-level *KRAS* G12C prevalence among H/L and NH-W patients using MSK-CHORD as the initial reference cohort and AACR GENIE after excluding the Memorial Sloan Kettering Cancer Center (MSK) contributing center. We focused on mutually exclusive lung adenocarcinoma and non–lung adenocarcinoma NSCLC strata, with NSCLC overall included only as a secondary descriptive analysis, and treated pan-cancer analyses as secondary context. Secondary analyses assessed broader and strict race/ethnicity definitions, tumor type–adjusted pan-cancer models, *KRAS* G12D, molecular context, MSK-CHORD sequential clinical models, and center-specific estimates with leave-one-center-out robustness in GENIE.

## Materials and Methods

### Data sources and study population

We performed a retrospective clinicogenomic study using the publicly available MSK-CHORD dataset accessed through cBioPortal (RRID: SCR_014555; refs. 13, 14) and AACR Project GENIE release 19.0-public data (11). MSK-CHORD served as the initial reference cohort. GENIE was analyzed as an independent comparison cohort after excluding cases from the MSK contributing center.

Available clinical, demographic, histologic, sequencing-assay, and somatic mutation fields were obtained from the corresponding clinical, sample-level, and mutation files for each cohort. Analyses were restricted to patients with race/ethnicity information sufficient to classify them as H/L or NH-W. Patients classified as other/unknown were excluded from H/L versus NH-W comparisons.

Primary lung analyses used mutually exclusive lung adenocarcinoma and non–lung adenocarcinoma NSCLC strata. NSCLC overall was evaluated secondarily and was not interpreted independently of its histologic composition. Pan-cancer analyses included all eligible sequenced patients and were treated as secondary context because *KRAS* G12C prevalence depends strongly on tumor type; tumor type–adjusted pan-cancer logistic models were reported separately.

### Race/ethnicity and clinical variables

Race and ethnicity were harmonized from available ethnicity and race fields. Patients were classified as H/L only when ethnicity explicitly indicated Spanish, Hispanic, Latino, or related H/L-origin categories and did not indicate non-Hispanic status. Patients were classified as NH-W only when race indicated White and ethnicity explicitly indicated non-Hispanic or non-Spanish status. White patients with missing, unknown, not-collected, surname-only, or otherwise ambiguous ethnicity were not inferred to be NH-W and were excluded from H/L versus NH-W comparisons. A sensitivity analysis assessed whether prevalence estimates were sensitive to the race/ethnicity classification definition; raw race and ethnicity values and classification decisions are summarized in Supplementary Table S1.

Clinical variables included age, sex, smoking status, stage, and sample type when available in the analysis files. Age was derived from available age-at-sequencing or current-age fields. Sex was harmonized as female or male when available. Smoking status was harmonized as never or ever when available; otherwise it was treated as unavailable. Stage was harmonized as stage IV or stage I to III when available; otherwise it was treated as unavailable. Sample type was harmonized as primary, metastatic, recurrence, or cell-free DNA/liquid when available. For descriptive summaries, metastatic and recurrent samples were grouped as metastatic/recurrent.

### Somatic mutation endpoints

Somatic mutation analyses used available mutation annotation files, prioritizing extended mutation files when available. Analyses were restricted to nonsilent variants, including missense, nonsense, frameshift deletion, frameshift insertion, splice-site, in-frame deletion, in-frame insertion, nonstop, and translation start-site mutations.

The primary genomic endpoint was *KRAS* G12C, defined as a nonsilent *KRAS* mutation with a protein-change annotation containing G12C. When explicit pathogenicity or oncogenicity annotations were available in the source mutation files, *KRAS* G12C analyses were restricted accordingly. A separate sensitivity analysis was restricted to canonical *KRAS* p.G12C missense variants. *KRAS* G12D was evaluated as an allele-specific comparator. Additional molecular endpoints included *KRAS*-any mutation and nonsilent mutations in *TP53*, *EGFR*, *STK11*, *KEAP1*, *SMARCA4*, and *NFE2L2*. Co-mutation analyses evaluated *STK11*, *KEAP1*, *TP53*, *SMARCA4*, and *STK11* or *KEAP1* within *KRAS*-mutant and *KRAS* G12C–positive subsets.

### Sequencing panel coverage

GENIE gene-level analyses used gene panel coverage information. For each gene-specific endpoint, patients were included in the denominator only when their sequencing panel covered the corresponding gene. *KRAS* G12C analyses in GENIE were restricted to patients sequenced with *KRAS*-covered panels. Group-specific coverage denominators by histologic subgroup are summarized in Supplementary Table S2.

MSK-CHORD gene endpoints were analyzed using the mutation calls provided in the public release, without gene-specific denominator reassignment.

### Patient-level aggregation

The primary analytic unit was the patient. For each disease stratum, sample-level records were collapsed to one patient-level record. A patient was considered positive for a genomic endpoint if any sample within the corresponding stratum harbored the alteration. For patients with multiple samples, categorical clinical variables were aggregated using prespecified rules that prioritized available known values. Age was summarized using the minimum available age within the stratum. Sample-level analyses were performed separately as sensitivity analyses (Supplementary Table S3).

### Statistical analysis

Primary analyses compared H/L and NH-W patients within each cohort and histologic stratum. Binary endpoints were summarized as number of events, analytic denominator, prevalence, and absolute prevalence difference in percentage points. Odds ratios (OR), 95% confidence intervals (CI), and *P* values were calculated for H/L versus NH-W comparisons. ORs below 1 indicate lower endpoint prevalence among H/L patients.

Unadjusted binary comparisons used Fisher exact tests. OR and CIs were estimated on the log-odds scale. For sparse or zero-cell comparisons, a 0.5 continuity correction was applied consistently to both the point estimate and CI, whereas Fisher exact tests were retained for *P* values.

Sequentially adjusted clinical models were restricted to MSK-CHORD. Logistic regression models used *KRAS* G12C as the outcome and H/L versus NH-W as the primary exposure. Sequential models adjusted for age and sex; age, sex, and smoking; age, sex, stage, and sample type; and age, sex, smoking, stage, and sample type. Models used complete-case data for the covariates included in each model. Multivariable covariate-adjusted models were not fit in GENIE because smoking and stage were not harmonized for GENIE in the analysis dataset.

GENIE center-level analyses were performed in the lung adenocarcinoma subset after excluding the MSK contributing center. Centers were included when they had at least 20 H/L patients with lung adenocarcinoma and 100 NH-W patients with lung adenocarcinoma (Supplementary Table S4). Center-specific *KRAS* G12C prevalence, absolute differences, *P* values, and ORs with 95% CIs when estimable were reported. Leave-one-center-out analyses were used to assess whether the pooled GENIE lung adenocarcinoma estimate depended on a single contributing center.

The primary endpoint was patient-level *KRAS* G12C prevalence in H/L versus NH-W patients in mutually exclusive lung adenocarcinoma and non–lung adenocarcinoma NSCLC strata across MSK-CHORD and GENIE after excluding the MSK contributing center. NSCLC overall, pan-cancer analyses, molecular context analyses, sample-level analyses, and center-level analyses were secondary or sensitivity analyses.

All data processing, statistical analyses, and figure generation were performed using Python version 3.11.10 with pandas version 2.3.3, NumPy version 1.26.4, SciPy version 1.16.3, statsmodels version 0.14.6, and Matplotlib version 3.10.7.

### Ethics statement

This study used publicly available, deidentified secondary clinicogenomic data and involved no direct patient contact or intervention. The original MSK-CHORD study was conducted under institutional review board (IRB)–approved protocols and obtained written informed consent from participants (13). AACR Project GENIE data are contributed under institution-specific IRB and consent procedures, including IRB-approved consent or waivers where applicable (11). No additional IRB approval or informed consent was required for this secondary analysis of publicly available deidentified data.

## Results

### Lower *KRAS* G12C prevalence in H/L patients is concentrated in lung adenocarcinoma

Using the strict race/ethnicity definition, the MSK-CHORD lung adenocarcinoma cohort included 267 H/L and 4,650 NH-W patients, and the non–lung adenocarcinoma NSCLC cohort included 84 H/L and 1,144 NH-W patients. In GENIE after MSK exclusion, the lung adenocarcinoma cohort included 732 H/L and 8,219 NH-W patients, and the non–lung adenocarcinoma NSCLC cohort included 203 H/L and 2,976 NH-W patients (Supplementary Table S5).

**Figure 1.**
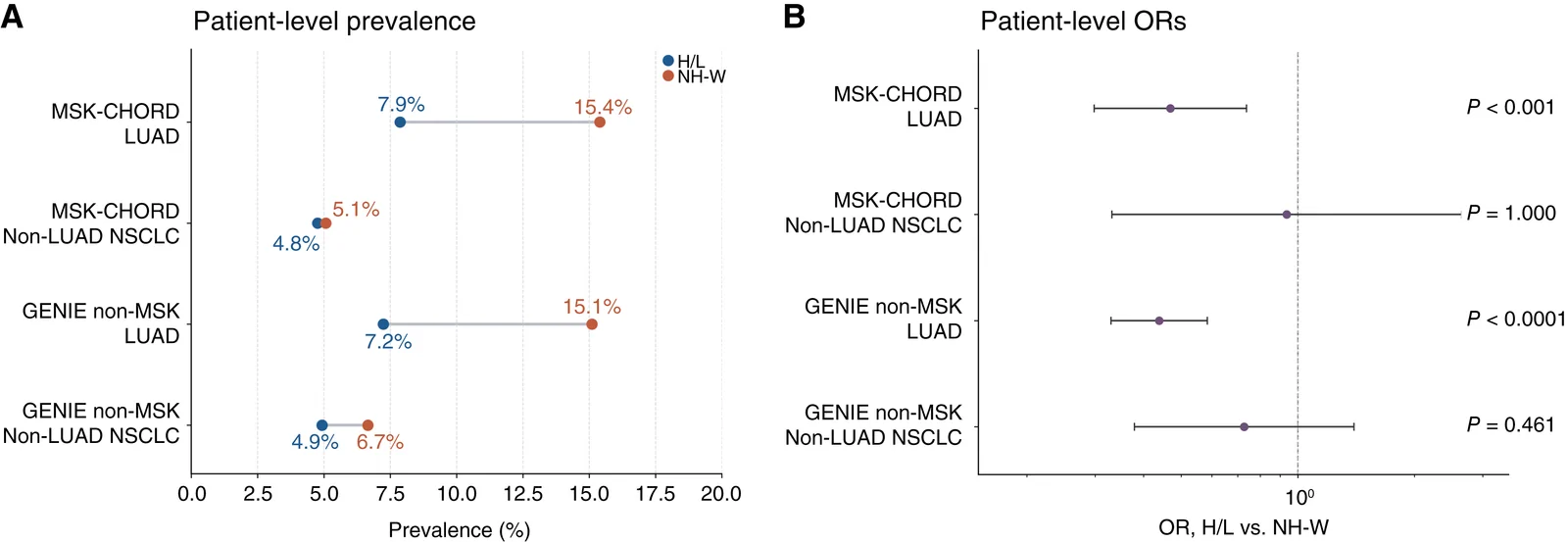
*KRAS* G12C prevalence in lung adenocarcinoma (LUAD) and non-LUAD NSCLC. **A,** Patient-level *KRAS* G12C prevalence in H/L and NH-W patients in mutually exclusive LUAD and non-LUAD NSCLC strata. **B,** Crude patient-level ORs comparing H/L vs. NH-W patients, with 95% CIs. OR < 1 indicates lower *KRAS* G12C prevalence in H/L patients. *P* values are from Fisher exact tests.

*KRAS* G12C prevalence was lower in H/L than NH-W patients with lung adenocarcinoma in both cohorts (Fig. 1A and B; Supplementary Table S5). In MSK-CHORD lung adenocarcinoma, the prevalence was 7.9% versus 15.4% (21/267 vs. 716/4,650; OR, 0.47; 95% CI, 0.30–0.74; *P* < 0.001). In GENIE lung adenocarcinoma after MSK exclusion, the prevalence was 7.2% versus 15.1% (53/732 vs. 1,241/8,219; OR, 0.44; 95% CI, 0.33–0.58; *P* < 0.0001).

In non–lung adenocarcinoma NSCLC, the corresponding differences were smaller and not statistically significant. In MSK-CHORD, the *KRAS* G12C prevalence was 4.8% in H/L patients and 5.1% in NH-W patients (4/84 vs. 58/1,144; OR, 0.94; 95% CI, 0.33–2.64; *P* = 1). In GENIE, the prevalence was 4.9% versus 6.7% (10/203 vs. 198/2,976; OR, 0.73; 95% CI, 0.38–1.40; *P* = 0.461). NSCLC overall retained a lower H/L prevalence in both cohorts, but this result was driven primarily by lung adenocarcinoma (Supplementary Fig. S1B).

### Strict race/ethnicity classification and tumor-type context

Under the strict classification, H/L status required explicit H/L ethnicity and NH-W status required White race with explicit non-Hispanic ethnicity. In MSK-CHORD, 1,569 patients were classified as H/L and 17,990 as NH-W; 1,005 patients meeting the broader classification definition but not the strict definition and 655 White patients with ambiguous ethnicity were excluded from H/L versus NH-W comparisons. At the race/ethnicity classification stage in GENIE after MSK exclusion, 10,282 patients were classified as H/L and 64,587 as NH-W; endpoint-specific analytic denominators were subsequently determined by disease stratum and sequencing eligibility, whereas 8,953 broader-definition NH-W patients with unknown or not-collected ethnicity were excluded (Supplementary Table S1A and S1B).

The lung adenocarcinoma association remained directionally consistent across the broader and strict race/ethnicity definitions. In MSK-CHORD lung adenocarcinoma, the ORs were 0.74 under the broader definition and 0.47 under the strict definition. In GENIE LUAD, the estimate was essentially unchanged (broader-definition OR, 0.45; strict OR, 0.44; Supplementary Fig. S1A). In secondary pan-cancer analyses, after adjustment for tumor type, the H/L versus NH-W OR remained below 1 in MSK-CHORD (OR, 0.64; 95% CI, 0.46–0.88; *P* = 0.006) and GENIE (OR, 0.68; 95% CI, 0.57–0.81; *P* < 0.0001; Supplementary Fig. S2B; Supplementary Table S6A), with model composition shown in Supplementary Table S6B.

### Clinical covariates and sequential adjustment in MSK-CHORD

**Figure 2.**
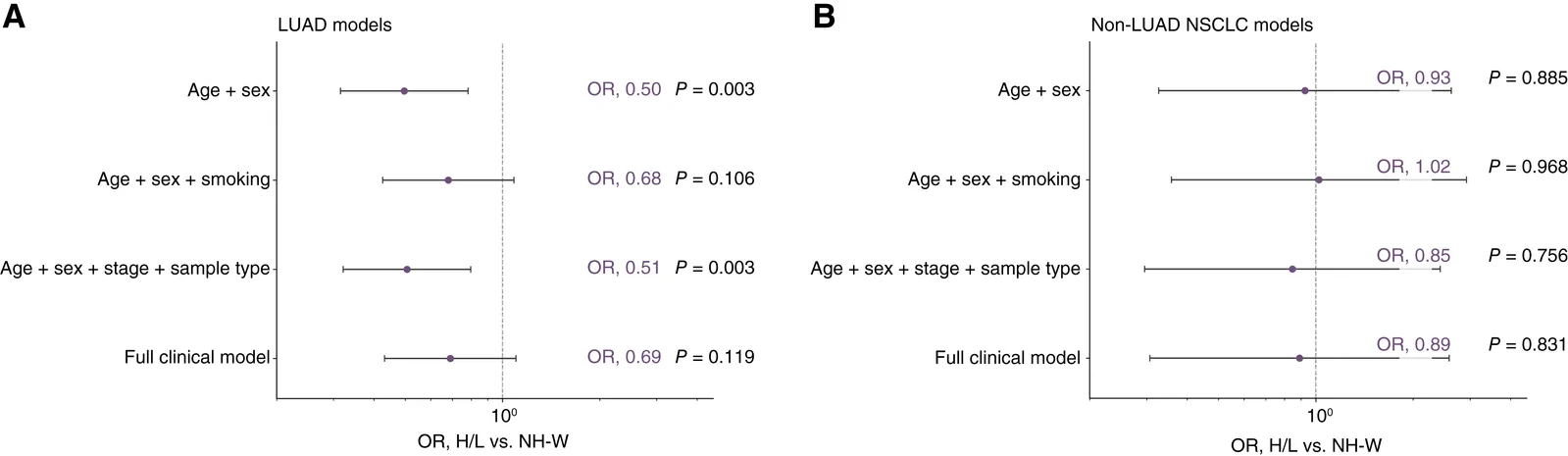
Clinical covariate adjustment in MSK-CHORD. **A,** MSK-CHORD lung adenocarcinoma (LUAD) adjusted logistic regression models. **B,** MSK-CHORD non-LUAD NSCLC adjusted logistic regression models. Figure labels show point estimates and *P* values. Models are limited to MSK-CHORD because smoking and stage variables were not harmonized for GENIE multivariable adjustment. These analyses describe changes in effect estimates after clinical covariate adjustment and should not be interpreted as a causal model of ethnicity.

Clinical adjustment in MSK-CHORD showed that the lung adenocarcinoma association was attenuated after smoking adjustment (Fig. 2A; Supplementary Table S7). In lung adenocarcinoma, the age- and sex-adjusted OR was 0.50 (95% CI, 0.32–0.78; *P* = 0.003). After adding smoking, the OR shifted toward 1 and was no longer statistically significant (OR, 0.68; 95% CI, 0.43–1.09; *P* = 0.106). The age-, sex-, stage-, and sample type–adjusted model yielded an OR of 0.51 (95% CI, 0.32–0.80; *P* = 0.003), whereas the full clinical model yielded an OR of 0.69 (95% CI, 0.43–1.10; *P* = 0.119).

### Molecular context, *KRAS* G12D, and co-mutation patterns

**Figure 3.**
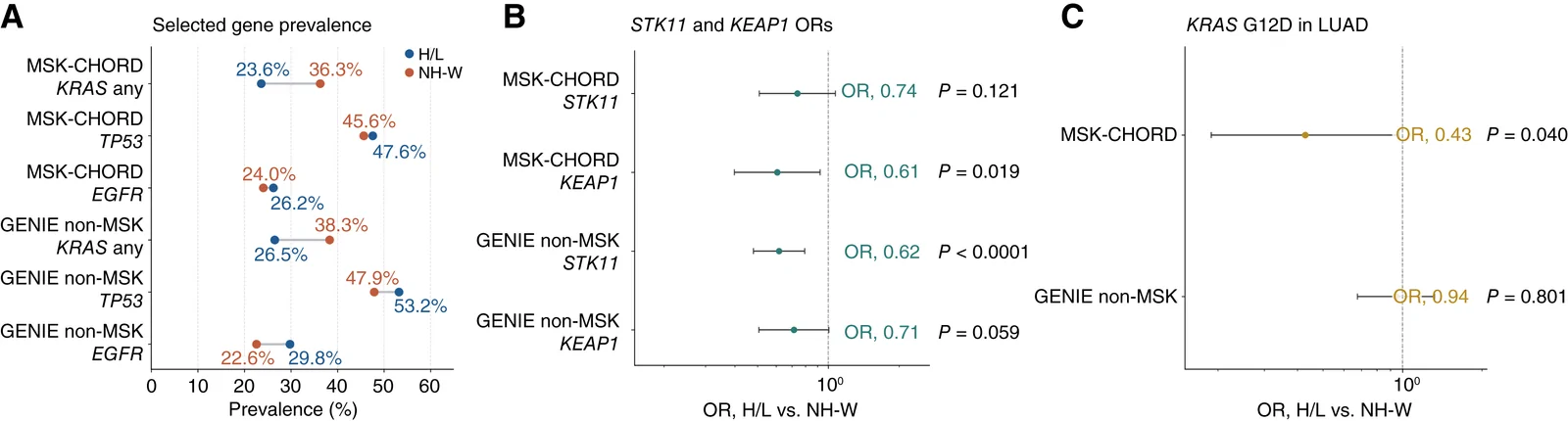
Selected genomic features in lung adenocarcinoma (LUAD). **A,** Selected LUAD molecular endpoint prevalence in H/L and NH-W patients. **B,***STK11* and *KEAP1* ORs in LUAD. **C,***KRAS* G12D ORs in LUAD. GENIE molecular analyses use coverage-aware denominators based on panel-gene coverage files.

In lung adenocarcinoma, *KRAS*-any alterations were less frequent in H/L than NH-W patients in both cohorts (Fig. 3A; Supplementary Table S8). In MSK-CHORD, the *KRAS*-any prevalence was 23.6% versus 36.3% (OR, 0.54; 95% CI, 0.41–0.72; *P* < 0.0001). In GENIE, the prevalence was 26.5% versus 38.3% (OR, 0.58; 95% CI, 0.49–0.69; *P* < 0.0001). *TP53* prevalence was similar in MSK-CHORD but higher in H/L patients in GENIE lung adenocarcinoma. *EGFR* alterations were not significantly different in MSK-CHORD lung adenocarcinoma but were more frequent in H/L patients in GENIE lung adenocarcinoma (29.8% vs. 22.6%; OR, 1.45; 95% CI, 1.23–1.72; *P* < 0.0001). The corresponding *STK11* and *KEAP1* OR estimates are shown in Fig. 3B.

*KRAS* G12D did not reproduce the same cross-cohort pattern as *KRAS* G12C. In MSK-CHORD lung adenocarcinoma, *KRAS* G12D prevalence was lower in H/L patients (2.2% vs. 5.1%; OR, 0.43; 95% CI, 0.19–0.97; *P* = 0.040). In GENIE lung adenocarcinoma, prevalence was similar between groups (5.3% vs. 5.6%; OR, 0.94; 95% CI, 0.68–1.32; *P* = 0.801; Fig. 3C; Supplementary Table S9).

Annotation status is summarized in Supplementary Table S10B. Results restricted to canonical *KRAS* p.G12C missense variants were concordant with the primary analysis (Supplementary Table S10A; Supplementary Fig. S2A).

Co-mutation analyses were exploratory because *KRAS* G12C–positive H/L subsets were small. In lung adenocarcinoma, the *STK11* or *KEAP1* composite among *KRAS* G12C–positive tumors was lower in H/L patients in MSK-CHORD (9.5% vs. 33%; OR, 0.21; 95% CI, 0.05–0.93; *P* = 0.030). In GENIE lung adenocarcinoma, *STK11* among *KRAS* G12C–positive tumors was lower in H/L patients (5.7% vs. 25.3%; OR, 0.18; 95% CI, 0.05–0.57; *P* < 0.001), whereas *TP53* was higher (60.4% vs. 41%; OR, 2.19; 95% CI, 1.25–3.85; *P* = 0.006; Supplementary Table S11).

### Lower *KRAS* G12C prevalence across GENIE contributing centers in lung adenocarcinoma

**Figure 4.**
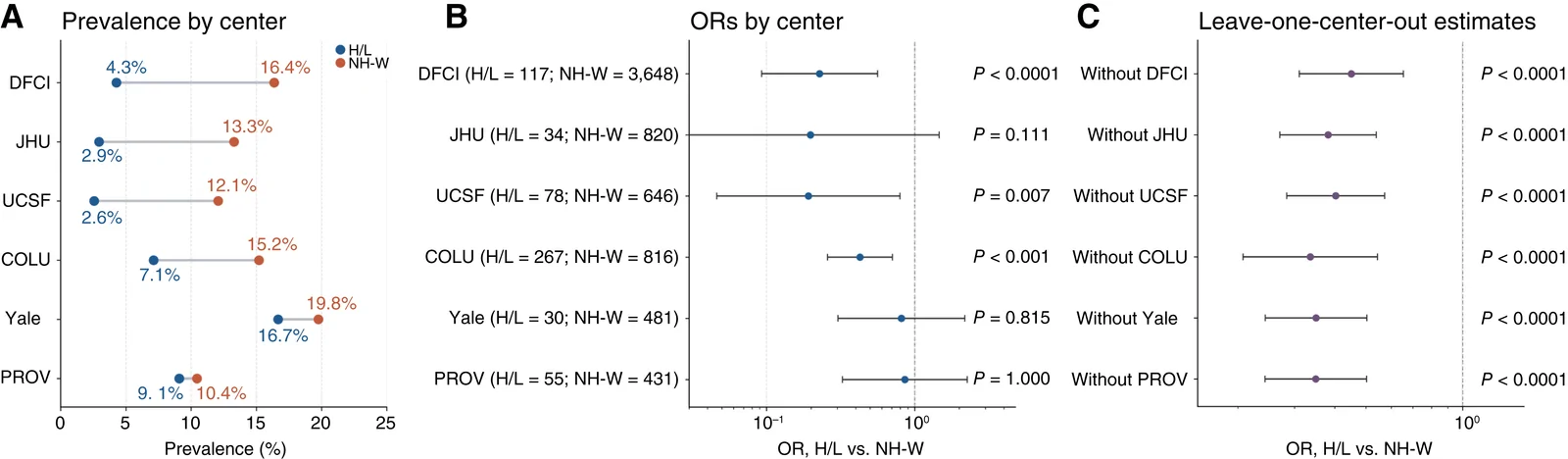
Center-specific *KRAS* G12C estimates in GENIE. **A,** Center-specific *KRAS* G12C prevalence in lung adenocarcinoma. DFCI, Dana-Farber Cancer Institute. **B,** Center-specific ORs comparing H/L vs. NH-W patients. **C,** Leave-one-center-out estimates for the pooled GENIE lung adenocarcinoma analysis. Centers were included using a predefined analytic threshold of H/L ≥ 20 patients with lung adenocarcinoma and NH-W ≥ 100 patients with lung adenocarcinoma. COLU, Herbert Irving Comprehensive Cancer Center, Columbia University; JHU, Johns Hopkins Sidney Kimmel Comprehensive Cancer Center; PROV, Providence Health and Services Cancer Institute; UCSF, University of California, San Francisco.

Six GENIE centers met the center-level threshold of at least 20 H/L patients with LUAD and 100 NH-W patients with LUAD after excluding the MSK contributing center (Supplementary Table S4A). *KRAS* G12C prevalence was lower in H/L than NH-W patients in each included center, although precision varied by center (Fig. 4A and B; Supplementary Table S12).

The largest absolute differences were observed at Dana-Farber Cancer Institute (4.3% vs. 16.4%; OR, 0.23; 95% CI, 0.09–0.56; *P* < 0.0001), JHU (2.9% vs. 13.3%; OR, 0.20; 95% CI, 0.03–1.46; *P* = 0.111), and UCSF (2.6% vs. 12.1%; OR, 0.19; 95% CI, 0.05–0.80; *P* = 0.007). Leave-one-center-out analyses retained a lower H/L prevalence estimate after excluding each eligible center individually (Fig. 4C; Supplementary Table S4B).

## Discussion

This study identified a lower prevalence of *KRAS* G12C among H/L patients compared with NH-W patients in lung adenocarcinoma across two large clinicogenomic cohorts. The pattern was present in MSK-CHORD and was also observed in GENIE after excluding the MSK contributing center. The mutually exclusive histology analysis showed that the difference was concentrated in lung adenocarcinoma and was not observed with comparable magnitude in non–lung adenocarcinoma NSCLC. The lung adenocarcinoma–specific finding is clinically relevant because *KRAS* G12C is enriched in this histology and is an established treatment-defining biomarker in NSCLC; *KRAS* G12C–directed therapy has been developed and studied in previously treated advanced *KRAS* G12C–mutated NSCLC (1, 5, 6). The broader *KRAS*-directed therapeutic landscape, including resistance biology and emerging combination strategies, has continued to expand beyond the initial *KRAS* G12C inhibitor studies (15).

Under strict race/ethnicity classification, H/L patients had approximately half the *KRAS* G12C prevalence of NH-W patients with lung adenocarcinoma in both cohorts; by contrast, non–lung adenocarcinoma NSCLC showed small, nonsignificant differences. This pattern indicates that the NSCLC overall result reflects the lung adenocarcinoma subset rather than a uniform difference across NSCLC histologies. MSK-CHORD and AACR GENIE are both designed for real-world clinicogenomic research, but their data structures and ascertainment patterns require careful analytic handling (12, 13).

The clinical relevance of the lung adenocarcinoma finding derives from the current role of *KRAS* G12C as an actionable NSCLC biomarker. Regulatory summaries and clinical trials have established sotorasib and adagrasib as *KRAS* G12C–directed therapies in previously treated advanced or metastatic NSCLC (5, 6, 16, 17). This study does not evaluate treatment receipt, access, response, progression-free survival (PFS), or overall survival (OS). It estimates how often the actionable alteration is detected in H/L and NH-W patients when real-world clinicogenomic datasets are analyzed using strict race/ethnicity classification, mutually exclusive histology strata, and patient-level denominators.

Published real-world data indicate that *KRAS* G12C prevalence in advanced NSCLC varies across geographic regions (18). Allele-specific *KRAS* G12C prevalence has also been reported to vary by race, sex, and cancer type. These data support estimating prevalence directly within the H/L and NH-W groups represented in MSK-CHORD and GENIE while avoiding extrapolation from geography-based series. Differences in country-of-origin composition, ancestry proportions, smoking patterns, referral populations, and local testing practices may contribute to variation in center-specific estimates. H/L classification in MSK-CHORD and GENIE should not be treated as a substitute for Latin American geography, genetic ancestry, country of origin, smoking history, or health-system context. Prior studies in H/L and Latin American lung cancer populations have shown that *EGFR* and *KRAS* frequencies can differ from those reported in predominantly NH-W cohorts, but they also highlight heterogeneity within H/L populations (9, 10, 19–21).

In MSK-CHORD, clinical models placed the lung adenocarcinoma difference in the context of smoking. After smoking was added to the lung adenocarcinoma model, the *KRAS* G12C OR shifted toward 1 and was no longer statistically significant. This is compatible with prior evidence linking smoking exposure with *KRAS* G12C in lung adenocarcinoma (22). This attenuation should not be read as a causal model of ethnicity. Rather, it indicates that smoking distribution is an important clinical correlate of the *KRAS* G12C prevalence difference observed in MSK-CHORD.

The molecular profile argues against broad technical underdetection of mutations in H/L tumors. In lung adenocarcinoma, *KRAS*-any alterations were also less frequent in H/L patients in both cohorts, consistent with the *KRAS* G12C result. *TP53* prevalence was similar in MSK-CHORD and higher in H/L patients in GENIE, whereas *EGFR* alterations were higher in H/L patients in GENIE. This distribution is consistent with prior reports that *EGFR* and *KRAS* alterations differ across population groups in lung adenocarcinoma (19, 20, 23). These endpoint-specific patterns are more informative than a single aggregate measure of mutation prevalence.

Co-mutation analyses added *KRAS*-context information. Among *KRAS* G12C–positive lung adenocarcinoma tumors, *STK11* or *KEAP1* co-mutation prevalence was lower in H/L patients in MSK-CHORD, and *STK11* prevalence was lower in H/L patients in GENIE. *TP53* prevalence among *KRAS* G12C–positive lung adenocarcinoma tumors was higher in H/L patients in GENIE. These genes are clinically relevant in *KRAS*-mutant NSCLC, and *STK11*/*KEAP1* alterations have been associated with distinct tumor immune features and less favorable outcomes in several advanced NSCLC settings (3, 24, 25). Because H/L *KRAS* G12C–positive subsets were small, these co-mutation results are exploratory and should not be interpreted as treatment-response or survival analyses.

Accurate prevalence estimation in real-world sequencing datasets requires patient-level aggregation, explicit race/ethnicity definitions, histology-specific strata, and coverage-aware denominators. The primary analysis used patient-level denominators, strict race/ethnicity classification, and mutually exclusive lung adenocarcinoma and non–lung adenocarcinoma NSCLC strata. GENIE molecular analyses used coverage-aware gene denominators. This matters because GENIE is a multi-institutional real-world clinicogenomic registry assembled across international cancer centers, and assay content differs across sequencing panels (11, 12). Pan-cancer analyses were retained as secondary context; after tumor-type adjustment, the H/L versus NH-W estimate remained below 1 in both cohorts. In GENIE, lower H/L prevalence was observed across eligible lung adenocarcinoma centers, although individual center estimates varied in precision.

Several limitations should be considered. Race and ethnicity were based on clinical annotations and should not be interpreted as genetic ancestry. The H/L category includes heterogeneous populations with different ancestry, geography, exposures, and health-system contexts. Requiring explicit ethnicity excludes patients with incomplete ethnicity data and can change cohort composition; therefore, broader-versus-strict analyses are provided. Smoking and stage were used in MSK-CHORD sequentially adjusted models, but comparable multivariable modeling was not performed in GENIE. MSK-CHORD gene-level analyses used the mutation calls provided in the public release without gene-specific denominator reassignment. The canonical *KRAS* p.G12C missense analysis was used as the allele-level sensitivity analysis consistently supported across both datasets. Treatment receipt, access to KRAS G12C inhibitors, response, PFS, and OS were not evaluated.

In summary, H/L patients had lower *KRAS* G12C prevalence than NH-W patients with lung adenocarcinoma across MSK-CHORD and GENIE after MSK exclusion. The non–lung adenocarcinoma NSCLC stratum showed only small, nonsignificant differences. In MSK-CHORD, smoking adjustment attenuated the lung adenocarcinoma association, supporting a clinicomolecular biomarker-yield interpretation rather than an ethnicity-intrinsic biological explanation. These findings inform expected *KRAS* G12C biomarker yield in H/L patients with lung adenocarcinoma and reinforce the need for patient-level, coverage-aware, histology-specific analyses when using real-world clinicogenomic data to study biomarker distribution across undercharacterized populations.

## Supplementary Material

Supplementary Figure S1 Figure S1. Ethnicity classification and NSCLC histologic composition

Supplementary FIgure S2 Supplementary Figure S2

Supplementary Table S1 Table S1. Race and ethnicity classification audit

Supplementary Table S2 Table S2. GENIE gene coverage by histologic subgroup

Supplementary Table S3 Table S3. Patient- and sample-level KRAS G12C estimates

Supplementary Table S4 Table S4. GENIE center eligibility and leave-one-out analyses

Supplementary Table S5 Supplementary Table S5. KRAS G12C prevalence by histologic subgroup

Supplementary Table S6 Table S6. Pan-cancer tumor-type-adjusted models

Supplementary Table S7 Table S7. MSK-CHORD sequentially adjusted models

Supplementary Table S8 Table S8. Selected genomic features in LUAD

Supplementary Table S9 Table S9. KRAS G12D prevalence by histologic subgroup

Supplementary Table S10 Table S10. Canonical KRAS p.G12C sensitivity and annotation status

Supplementary Table S11 Table S11. Co-mutations in KRAS-positive LUAD

Supplementary Table S12 Table S12. GENIE center-specific LUAD estimates

## Data Availability

The MSK-CHORD dataset is publicly available through cBioPortal (RRID: SCR_014555). AACR Project GENIE release 19.0-public is available through the GENIE data portal and cBioPortal, subject to the access terms of each resource. The analysis used deidentified secondary data only. Derived tables, figures, and supporting source data are provided with the article and its supplementary materials.
